# Prophylactic infusion of phenylephrine increases the median effective dose of intrathecal hyperbaric bupivacaine in cesarean section: A prospective randomized study

**DOI:** 10.1097/MD.0000000000011833

**Published:** 2018-08-10

**Authors:** Yin-Fa Zhang, Fei Xiao, Wen-Ping Xu, Lin Liu

**Affiliations:** Department of Anesthesia, Jiaxing University Affiliated Women and Children Hospital, Jiaxing Maternity and Child Care Hospital, Zhejiang, China.

**Keywords:** bupivacaine, cesarean section, combined spinal and epidural, phenylephrine

## Abstract

**Background:**

Phenylephrine infusion to prevent spinal-induced hypotension can attenuate cephalic spread of intrathecal bupivacaine. Therefore, we suspected the intrathecal dose requirement for bupivacaine may differ when using phenylephrine infusion to prevent spinal-induced hypotension in cesarean section. We designed a prospective, randomized study to determine the ED_50_ of hyperbaric bupivacaine for cesarean section under combined spinal-epidural anesthesia in healthy parturients with and without prophylactic phenylephrine infusion to prevent spinal-induced hypotension.

**Methods:**

Sixty healthy parturients rated American Society for Anesthesiology status I/II undergoing elective cesarean section were enrolled in this study, which was conducted July 2016 to February 2017 in the labor and delivery department of Jiaxing University Affiliated Women and Children Hospital. After enrollment, patients were randomized into 2 groups of 30 by blinded opaque envelopes sorted by computer-generated random allocation. Solutions were prepared by an anesthesiologist not involved in outcome measurement. Patients and anesthesiologists collecting data were blinded to group allocation. Group P (phenylephrine group) parturients received prophylactic infusion of phenylephrine at the time of intrathecal injection. Group S (saline group) parturients receive the same volume of saline. Doses of intrathecal bupivacaine for each patient were determined using an up-down allocation method; initial dose was 7 mg. Effective dose was defined as bilateral T6 or above sensory block level achieved within 10 minutes of intrathecal drug administration and no additional epidural lidocaine required for intraoperative pain. The Dixon and Massey formula was used to calculate ED_50_ values.

**Results:**

The ED_50_ values for hyperbaric bupivacaine were 7.0 mg (95% confidence interval [CI]: 6.6–7.4 mg) and 4.9 mg (95% CI: 4.4–5.4 mg) for groups P and S, respectively (*P* < .001). There were significant differences in incidence of hypotension and pH of umbilical arterial blood between groups S and P (60% vs 10%, *P* = .04 and 7.31 ± 0.04 vs 7.28 ± 0.06, *P* = .003, respectively).

**Conclusion:**

The ED_50_ of intrathecal hyperbaric bupivacaine is higher when phenylephrine infusion is used to prevent spinal-induced hypotension than when it is not used.

## 1 Introduction

Spinal anesthesia is regarded as one of the most acceptable methods of anesthesia for cesarean section because of its rapid onset and precise effect.^[1]^ However, the procedure is associated with a high incidence of hypotension, which is related to maternal and infant morbidity and mortality, if no prophylactic strategies are used to prevent spinal-induced hypotension; these strategies include uterine displacement, intravenous fluid pre-load or co-load, and the use of vasopressors.^[2]^

Recent studies have shown that prophylactic infusion of phenylephrine has more advantages in prevention of spinal-induced hypotension and intraoperative nausea and vomiting than does bolus administration.^[3,4]^ Interestingly, studies have shown that phenylephrine infusion for prevention of spinal-induced hypotension can attenuate cephalic spread of intrathecal bupivacaine.^[5,6]^ However, to our knowledge, this is the first study to investigate whether use of phenylephrine infusion in cesarean section affects the dose of spinal anesthetic required. We hypothesized that a higher dose of intrathecal local anesthetic may be needed when phenylephrine infusion is used to prevent spinal-induced hypotension in cesarean section under spinal anesthesia. To test our hypothesis, we designed a prospective study to investigate whether prophylactic infusion of phenylephrine increased the median effective dose (ED_50_) of spinal hyperbaric bupivacaine, when combined with low-dose sufentanil as an intrathecal adjuvant, in parturients undergoing cesarean section under combined spinal-epidural anesthesia (CSEA).

## 2 Methods

### 2.1 Trial design

This study was approved by the Institutional Review Board of our hospital (Jiaxing University Affiliated Women and Children Hospital, Zhejiang, China). All patients provided written informed consent. We registered this study with the Chinese Clinical Trial Registry (www.chictr.org.cn; registration number: ChiCTR-IIR-17011824). We designed a prospective, randomized study to determine the ED_50_ of hyperbaric bupivacaine for cesarean section under CSEA in healthy parturients with and without use of prophylactic phenylephrine infusion to prevent spinal-induced hypotension. This study followed the CONSORT Statement guidelines.

### 2.2 Participants

Sixty healthy patients with American Society of Anesthesiologists status I or II who were undergoing elective cesarean section were enrolled in this study, which was conducted between July 2016 and February 2017 in the labor and delivery department of Jiaxing University Affiliated Women and Children Hospital. Exclusion criteria were as follows: patients with body mass index >35 kg/m^2^; any contraindications to local anesthesia, including coagulopathy and local or general infection; obstetrical issues such as preeclampsia, ruptured membranes, gestational age <37 weeks, cesarean section history, early or active labor, or intrauterine growth restriction; and coexisting diseases, including diabetes or hypertension.

### 2.3 Interventions

There was no premedication. All parturients had venipuncture with an 18-G intravenous (IV) cannula through an arm vein in the preoperative preparation room, and 37°C lactated Ringer solution was slowly injected to keep the vein open. Baseline arterial blood pressure was the mean of 3 consecutive readings at 3-minute intervals during which the systolic blood pressure (SBP) did not vary by >10% from the average value in the preparation room. After arriving in the operating theater, standard monitoring measurements were consecutively recorded and included noninvasive blood pressure (NIBP), heart rate (HR), peripheral oxygen saturation (SpO_2_), and electrocardiogram. The mixed intrathecal local anesthetic (0.5% bupivacaine + sufentanil [5 μg] + 0.5 mL 10% dextrose, diluted with saline to a total volume of 3 mL; an insulin syringe [1 mL] was used to measure volumes [<1 mL]) was prepared under sterile conditions.

With all the patients in a left lateral position, anesthesiologists (WPX, LL, and YFZ) performed the CSEA technique using the needle-through-needle method (27-G spinal needle with pencil tip/18-G Tuohy needle). The epidural space was established at the L3-L4 interspace along the midline using the loss-of-resistance technique to a volume of <2 mL air. After ascertaining the emergence of cerebrospinal fluid (CSF), the study solutions were injected cephalically over 15 seconds through the spinal needle. An epidural catheter was passed through the Tuohy needle and placed 3 to 4 cm into the epidural space after the intrathecal injection. Theoretically, no local anesthetic or saline entered epidural space through the catheter. Each parturient was then placed on her left side at a 15° angle to supine position.

At the time of spinal injection, patients in group P received a prepared solution of 50 mL phenylephrine (0.2 mg/mL); patients in group S received a prepared solution of 50 mL saline. The infusion rate was adjusted according to variations in SBP. If the SBP was at or below baseline (not <80% of baseline), infusion continued. If the SBP decrease exceeded 20% of baseline, IV phenylephrine 100 μg was injected to treat hypotension. If SBP increased more than 20% of baseline or over 140 mm Hg, which was considered hypertension, infusion was discontinued; infusion was restarted when SBP decreased below 120% of baseline. Bradycardia was defined as HR < 50 beats per minute (bpm); if it was accompanied by hypotension, bradycardia was treated with IV atropine 0.5 mg; if bradycardia was not accompanied by hypotension, infusion was discontinued and was restarted when HR > 50 bpm. A co-load of 500 mL lactated Ringer solution was administered over a period of 15 to 20 minutes.

Each parturient's bupivacaine dose for intrathecal injection for was determined by the up-down approach.^[7]^ For each groups, the intrathecal dose for each patient was determined by the response (effective or ineffective) of the previous patient, with an initial dose of intrathecal hyperbaric bupivacaine 7 mg and a variable dose of 0.5 mg. In brief, if there was an effective response to the current dose, a lower dose (0.5 mg less than the current dose) was administered to the next patient. Conversely, if there was an ineffective response to the current dose, a higher dose (0.5 mg more than the current dose) was administered to the next patient.

### 2.4 Outcomes

The primary outcome in the current study was effective or ineffective dose of spinal bupivacaine anesthesia. An effective dose was defined according to our previous reports^[8,9]^ as the sensory block level achieved at T6 (bilateral) within 10 minutes of intrathecal injection; no 2% lidocaine was administered to patients via epidural catheter to rescue the induction of anesthesia or to manage pain during surgery. Otherwise, a case was considered ineffective spinal anesthesia, and 5 mL 2% lidocaine was administrated into the epidural space to rescue induction of anesthesia or to manage the pain during surgery, with the procedure repeated at 5-minute intervals as necessary. If additional epidural rescue doses were also ineffective, general anesthesia was administered. The characteristics of spinal anesthesia and the side-effects of spinal anesthesia were recorded as secondary outcomes.

After the intrathecal injection, NIBP and HR were measured and recorded before newborn delivery and were then recorded them at 5-minute intervals before completion of surgery.

The sensory block level was gently checked bilaterally via a 17-G needle, along the mid clavicular line. The lower sensory block level was considered the final sensory block level if it was not bilateral. The period between intrathecal injection and achievement of T10 sensory block was regarded as the onset time of sensory block. The period from sensory block onset to 2-segment regress to pinprick was defined as the duration of sensory block. Bromage score^[10]^ was used to assess motor block (0 = no motor block; 1 = cannot flex the hip; 2 = cannot flex the knee; and 3 = cannot flex the ankle). Characteristics of sensory and motor block were evaluated at 2-minute intervals after intrathecal injection and at 10-minute intervals until the completion of surgery. The total dose of phenylephrine used during surgery was recorded.

The visual analog scale (0 denotes pain, and 10 denotes the most undesirable pain) was used to appraise operative pain at different points of the surgical procedure, such as skin incision, fetal section, and peritoneal closure.

Side effects such as hypotension, hypertension, bradycardia, nausea and vomiting, shivering, and pruritus were assessed in this study. Blood gas analysis of umbilical arterial blood and the neonatal Apgar score were also assessed.

### 2.5 Sample size

According to Tyagi et al^[11]^ and our prior study,^[12]^ the sample size is regarded as adequate once 6 pairs of reversals of sequence are achieved when using the up-down method to determine the ED_50_ of intrathecal bupivacaine. Each group achieved more than 6 pairs of reversals of sequence after 30 patients enrolled in this study. The ED_50_ for each group was calculated with the Dixon and Massey formula.^[7]^ To test a difference of 2 mg in the dose requirement of intrathecal bupivacaine (ED_50_) with an α error of 0.05 and a test power of 90%, at least 21 patients were needed for each group. Therefore, 30 patients are sufficient for each group.

### 2.6 Randomization

After enrollment, patients were randomized by means of blinded opaque envelopes that had been sorted by computer-generated random allocation (Microsoft Excel; Microsoft Corp, Redmond, WA). Sixty parturients were randomized into 2 groups according whether prophylactic phenylephrine infusion was used to prevent spinal-induced hypotension: group S (saline group) (n = 30; no prophylactic phenylephrine infusion) and group P (phenylephrine group) (n = 30, with prophylactic phenylephrine infusion).

### 2.7 Blinding

The solutions were prepared by an anesthesiologist not involved in outcome measurement. CSEA was performed by an anesthesiologist who remained blinded to patient groups. Patients and the anesthesiologists collecting data were all blinded to group allocation.

### 2.8 Statistical methods

Demographic data including age, weight, and height, together with gestation age, duration of surgery, onset time to T10, and duration of sensory block, were described as mean ± standard deviation and analyzed via Student *t* test. Differences in ED_50_ were also analyzed via Student *t* test. Incidence of side effects and percentage of patients with Bromage score of 1 or 2 were analyzed with Chi-squared test; maximum sensory level and rescued lidocaine were presented as median (range) and analyzed with Mann–Whitney *U* test. Mean arterial pressures (MAPs) at different time points were evaluated between groups by repeated analysis of variance (Sidak multiple comparisons test). Kolmogorov–Smirnov test was applied to determine normal distribution. Statistical analysis was accomplished by using Graphpad Prism 5 (Version 5.01; GraphPad Software, San Diego, CA). *P* < .05 was considered statistically significant.

## 3 Results

**Figure 1:**
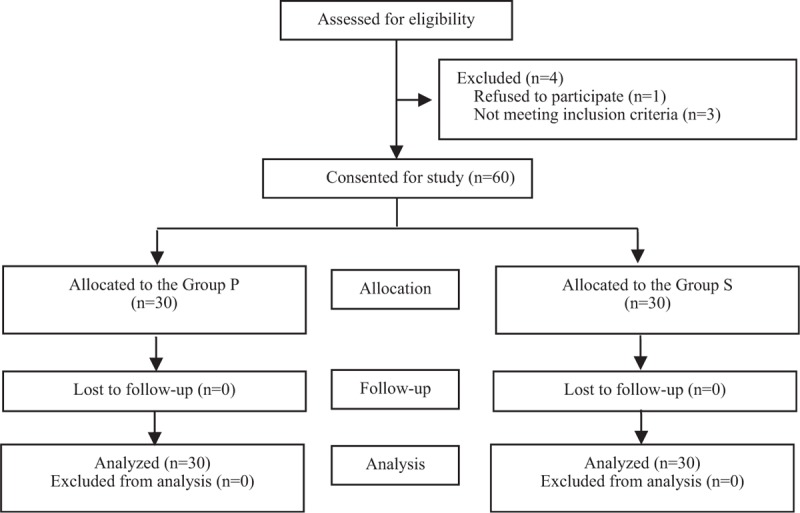
CONSORT flow diagram.

Sixty-four parturients were recruited for this study before grouping, and 60 of them were allocated into 2 groups (n = 30 for each group) and eventually completed the final analysis. (Fig. 1) One parturient refused to participate, and 3 parturients did not meet the inclusion criteria for the study. Demographic and obstetric characteristics and duration of surgery were similar between the2 groups (Table 1).

**Table 1:**
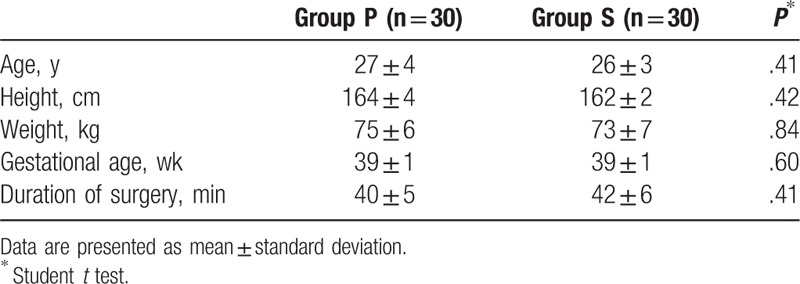
The characteristics of the demographic and obstetric and duration of surgery.

**Figure 2:**
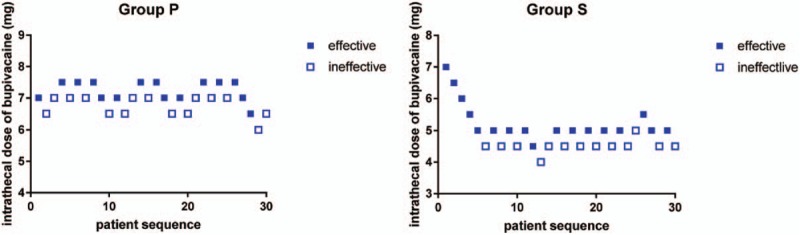
Response to corresponding dose.

The ED_50_ values for intrathecal bupivacaine were 7.0 mg (95% confidence interval [CI]: 6.6–7.4 mg) and 4.9 mg (95% CI: 4.4–5.4 mg) for groups P and S, respectively. The ED_50_ of bupivacaine was higher in group P than in group S (*P* < .001). Results of the up-down allocation approach for intrathecal dosing of bupivacaine are presented in Figure 2. There were 15 parturients in group P and 13 parturient in group S who required epidural 2% lidocaine to complement the induction of spinal anesthesia. Use of epidural rescue 2% lidocaine was not different between groups P and S (5 mL [5–10 mL] vs 5 mL [5–10 mL]) (*P* = .23).

Characteristics of subarachnoid block in parturients with effective anesthesia are presented in Table 2. There were no significant differences in maximum sensory block level (T5 [4–6] vs T5 [3–6], *P* = .45), onset time (4.7 ± 1.2 vs 4.5 ± 1.4, *P* = .26), or duration of spinal anesthesia (47 ± 19 vs 44 ± 16, *P* = .40). The percentage of parturients who scored 1 or 2 points on the Bromage scale was not statistically significance (*P* = .80).

**Table 2:**
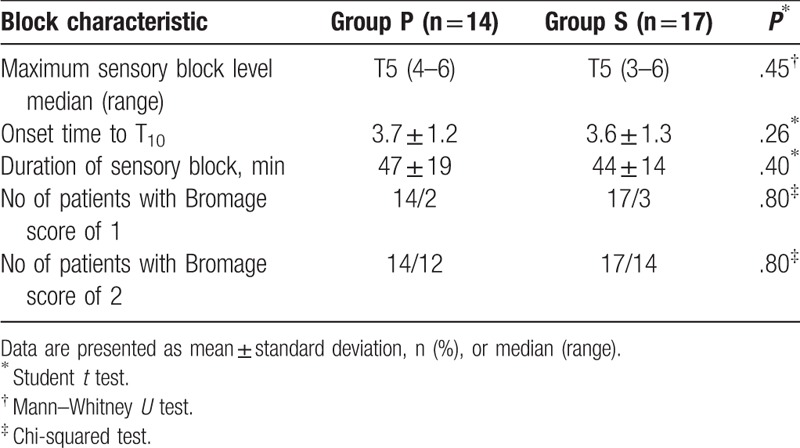
Characteristic of spinal anesthesia in “effective anesthesia” patients.

**Figure 3:**
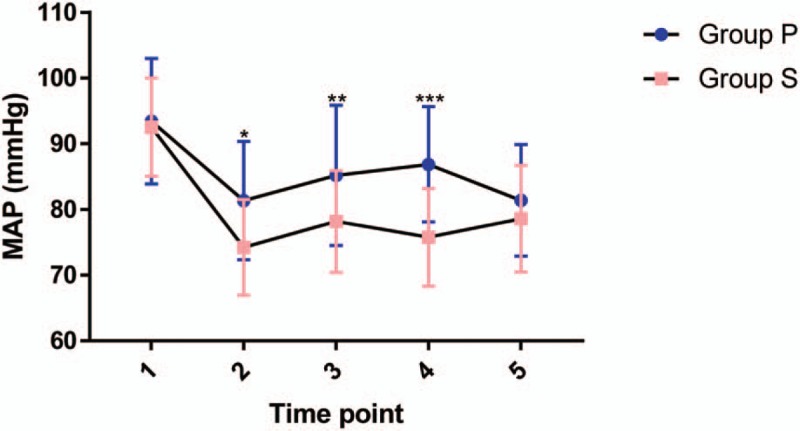
Intraoperative mean arterial pressure (MAP). Compared with group S, MAP in group P at time points 2, 3, and 4 was significantly higher (*P* < .05). 1 = baseline MAP, 2 = 5 min after SA, 3 = 10 min after SA, 4 = 20 min after SA, 5 = 30 min after SA, SA = spinal anesthesia. ^∗^*P* = .007, ^∗∗^*P* = .008, ^∗∗∗^*P* < .001 (Sidak multiple comparisons test).

Intraoperative MAP was higher at time points 2, 3, and 4 in group P than in group S (Fig. 3). The total dose of phenylephrine administered in group P was significant higher than that administered in group S (735 [380, 1080] vs 0 [0, 700], *P* *<* .001). There were no significant differences in the percentage of side effects (hypertension, bradycardia, nausea and vomiting, shivering, and pruritus) between parturients in the 2 groups (Table 3). There was significant difference in the incidence of hypotension between group S and group P (60% vs 10%, *P* = .04). The pH of umbilical arterial blood in group P was significantly higher than that in group S (7.31 ± 0.04 vs 7.28 ± 0.06, *P* = .003).

**Table 3:**
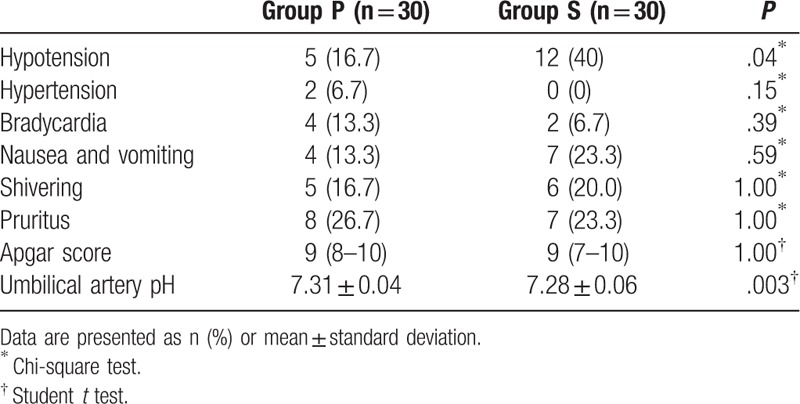
Side effects of anesthesia and neonatal Apgar score and umbilical arterial pH.

## 4 Discussion

We determined that the ED_50_ values of spinal bupivacaine (hyperbaric) along with sufentanil 5 μg for cesarean section with or without ongoing phenylephrine infusion to prevent spinal-induced hypotension were 7.0 mg (95% CI: 6.6–7.4 mg) and 4.9 mg (95% CI: 4.4–5.4 mg), respectively. Our results suggested that when we adopted the strategy of prophylactic continuous infusion of phenylephrine to prevent spinal-induced hypotension during cesarean section, a higher ED_50_ of intrathecal bupivacaine may be required. To our knowledge, this is the first study to compare the effect of preventive continuous injection of phenylephrine on the dose requirement for intrathecal hyperbaric bupivacaine.

Pregnancy can result in the hemangiectasis in the epidural space resulting from increased intraabdominal pressure that can cause a reduction in lumbar CSF volume.^[5]^ This causes a reduction in the intrathecal dosing requirement of local anesthetic or augments its spinal spread. However, the prophylactic infusion of phenylephrine to prevent postspinal hypotension may contract the veins in epidural space. It may abate the effects of epidural vein engorgement by replacing the CSF in the lumbar area, subsequently offsetting the pregnancy-induced decrease in intrathecal dose requirements. This could be the first mechanism to explain the results in our study. A second possible mechanism is that phenylephrine, as well as epinephrine, delays achievement of the spinal block. In the present study, effective spinal anesthesia was defined as achievement of sensory block at T6 (bilateral) within 10 minutes of intrathecal injection. In this clinical trial, waiting longer may lead to increased success in spinal induction, but may also result in increased surgical failure. However, further studies are needed. Previous studies have reported that IV infusion of phenylephrine can affect the spread of spinal local anesthetics (hyperbaric bupivacaine or plain levo-bupivacaine) by 2 mechanisms.^[6,13]^ However, the clinical significance of this finding remains unknown. In the present study, we used the up-down allocation method to determine that a higher dose was needed when phenylephrine infusion was used to prevent postspinal hypotension.

The ED_50_ of intrathecal bupivacaine has been assessed in parturients in several studies. Tyagi et al reported that, together with fentanyl 20 μg, the ED_50_ of spinal hyperbaric bupivacaine was 4.7 mg in normotensive and preeclamptic patients during cesarean section.^[11]^ Our previous study indicated that the ED_50_ values of intrathecal hyperbaric bupivacaine (along with 5 μg sufentanil) with or without magnesium sulfate were 4.9 or 4.7 mg, respectively, in cesarean section.^[12]^ These results were similar to those seen in this study. However, in a dose–response study conducted by Ginosar et al, the ED_50_ of intrathecal hyperbaric bupivacaine (mixed with morphine 200 μg and fentanyl 10 μg) was 7.6 mg in cesarean section.^[14]^ Several factors may contribute to this discrepancy. First, different methodologies were applied in the 2 studies. The up-down approach was used in our study, while regression analysis was utilized in their clinical trial. Different methods for determining ED_50_ or ED_95_ would produce differences in results. Second, different positions of patients’ heads may lead to different results. In our study, patients were placed in the left lateral position. Pregnant women with hips wider than the shoulders may end up in a head-down position.^[15]^ In the Ginosar et al study,^[14]^ CSEA was performed with patients in a sitting position. Therefore, the effect of gravity inevitably caused different results in the 2 studies. Finally, other factors, including duration of surgery, surgical technique (such as exteriorization of the uterus), use of different opioids, and patient height, can also affect results.

Figure 3 clearly shows that prophylactic continuous infusion of phenylephrine (25 μg/min) improved the hemodynamic stability of parturients undergoing cesarean section under CSEA. The incidence of hypotension was decreased by phenylephrine infusions in group P (16.7% vs 40%). Although there was no significant difference in nausea and vomiting between the 2 groups, the incidence of nausea and vomiting was 10% lower in the phenylephrine infusion group than in the saline infusion group. Our results reinforced previous findings that prophylactic infusion of phenylephrine provides better prevention of spinal-induced hypotension and reduction of nausea and vomiting than does bolus administration.

There are limitations to the current clinical trial. First, ED_95_, which is more relevant to clinical experience than ED_50_, may be a better choice for determining the impact of phenylephrine infusion on dose requirements of intrathecal hyperbaric bupivacaine in the present study. Nevertheless, we choose ED_50_ as an assessment standard for the following reasons: The up-down method is the classic means for evaluating the efficiency of a drug, and it has the advantage of reducing the sample size needed^[7]^; CSEA technology was utilized in this study. The epidural catheter allows subsequent topping-up to rescue an ineffective anesthesia. Further studies are needed to determine the ED_95_ of intrathecal hyperbaric bupivacaine with prophylactic infusion to prevent postspinal hypotension. Second, sufentanil was used as the intrathecal adjuvant in this study, and it can increase the effectiveness of intrathecal bupivacaine. It is possible that not using an opioid led to more precise results. Third, the additional dose of phenylephrine in group S may have affected the outcomes of the study. However, the hypothesis of the present study is that a higher dose of intrathecal local anesthetic may be needed when phenylephrine infusion was used to prevent spinal-induced hypotension in cesarean section under spinal anesthesia. Our results affirmed our hypothesis. Therefore, we believed that the effect of the additional dose of phenylephrine did not substantially affect our results, although it may have had a slight impact on the primary results.

In conclusion, the ED_50_ of intrathecal hyperbaric bupivacaine for patients undergoing cesarean section with prophylactic phenylephrine infusion to prevent spinal-induced hypotension is higher than the ED_50_ when phenylephrine infusion is not utilized.

## Acknowledgments

The authors most honestly appreciate all colleagues in the department of anesthesiology and theater of Jiaxing University Affiliated Women and Children Hospital for their assistance in the present study.

## Author contributions

**Data curation:** Ping Wen Xu, Lin Liu, Fa Yin Zhang.

**Investigation:** Fa Yin Zhang.

**Methodology:** Fei Xiao, Lin Liu, Fa Yin Zhang.

**Writing – original draft:** Fei Xiao.

**Writing – review & editing:** Fei Xiao.

## References

[1] Gizzo S Noventa M Fagherazzi S Update on best available options in obstetrics anaesthesia: perinatal outcomes, side effects and maternal satisfaction. Fifteen years systematic literature review. Arch Gynecol Obstet 2014;290:21–34. 24659334 10.1007/s00404-014-3212-x

[2] Ngan Kee WD Khaw KS Lee BB A dose response study of prophylactic intravenous ephedrine for the prevention of hypotension during spinal anesthesia for cesarean delivery. Anesth Analg 2000;90:1390–5. 10825326 10.1097/00000539-200006000-00024

[3] Ngan Kee WD Khaw KS Ng FF Prophylactic phenylephrine infusion for preventing hypotension during spinal anesthesia for cesarean delivery. Anesth Analg 2004;98:815–21. 14980943 10.1213/01.ane.0000099782.78002.30

[4] das Neves JF Monteiro JA de Almeida JR Phenylephrine for blood pressure control in elective cesarean section: therapeutic versus prophylactic doses. Rev Bras Anestesiol 2010;60:391–8. 20659611 10.1016/S0034-7094(10)70048-9

[5] Cooper DW Jeyaraj L Hynd R Evidence that intravenous vasopressors can affect rostral spread of spinal anesthesia in pregnancy. Anesthesiology 2004;101:28–33. 15220768 10.1097/00000542-200407000-00007

[6] Ngan Kee WD Lee A Khaw KS A randomized double-blinded comparison of phenylephrine and ephedrine infusion combinations to maintain blood pressure during spinal anesthesia for cesarean delivery: the effects on fetal acid-base status and hemodynamic control. Anesth Analg 2008;107:1295–302. 18806043 10.1213/ane.0b013e31818065bc

[7] Dixon WJ Staircase bioassay: the up-and-down method. Neurosci Biobehav Rev 1991;15:47–50. 2052197 10.1016/s0149-7634(05)80090-9

[8] Xiao F Xu WP Zhang XM ED50 and ED95 of Intrathecal bupivacaine coadministered with sufentanil for cesarean delivery under combined spinal-epidural in severely preeclamptic patients. Chin Med J (Engl) 2015;128:285–90. 25635420 10.4103/0366-6999.150083 PMC4837855

[9] Xiao F Xu WP Zhang YF The dose-response of intrathecal ropivacaine co-administered with sufentanil for cesarean delivery under combined spinal-epidural anesthesia in patients with scarred uterus. Chin Med J (Engl) 2015;128:2577–82. 26415793 10.4103/0366-6999.166036 PMC4736859

[10] Bromage PR A comparison of the hydrochloride and carbon dioxide salts of lidocaine and prilocaine in epidural analgesia. Acta Anaesthesiol Scand Suppl 1965;16:55–69. 5322004 10.1111/j.1399-6576.1965.tb00523.x

[11] Tyagi A Kakkar A Kumar S ED50 of hyperbaric bupivacaine with fentanyl for cesarean delivery under combined spinal epidural in normotensive and preeclamptic patients. Reg Anesth Pain Med 2012;37:40–4. 22030721 10.1097/AAP.0b013e318233c5f5

[12] Xiao F Xu W Feng Y Intrathecal magnesium sulfate does not reduce the ED50 of intrathecal hyperbaric bupivacaine for cesarean delivery in healthy parturients: a prospective, double blinded, randomized dose-response trial using the sequential allocation method. BMC Anesthesiol 2017;17:8. 28095795 10.1186/s12871-017-0300-z PMC5240204

[13] Cooper DW Gibb SC Meek T Effect of intravenous vasopressor on spread of spinal anaesthesia and fetal acid-base equilibrium. Br J Anaesth 2007;98:649–56. 17347185 10.1093/bja/aem056

[14] Ginosar Y Mirikatani E Drover DR ED50 and ED95 of intrathecal hyperbaric bupivacaine coadministered with opioids for cesarean delivery. Anesthesiology 2004;100:676–82. 15108985 10.1097/00000542-200403000-00031

[15] Khaw KS Ngan Kee WD Wong M Spinal ropivacaine for cesarean delivery: a comparison of hyperbaric and plain solutions. Anesth Analg 2002;94:680–5. 11867397 10.1097/00000539-200203000-00037

